# Tumor-targeting Salmonella typhimurium A1-R regresses an osteosarcoma in a patient-derived xenograft model resistant to a molecular-targeting drug

**DOI:** 10.18632/oncotarget.14040

**Published:** 2016-12-20

**Authors:** Takashi Murakami, Kentaro Igarashi, Kei Kawaguchi, Tasuku Kiyuna, Yong Zhang, Ming Zhao, Yukihiko Hiroshima, Scott D. Nelson, Sarah M. Dry, Yunfeng Li, Jane Yanagawa, Tara Russell, Noah Federman, Arun Singh, Irmina Elliott, Ryusei Matsuyama, Takashi Chishima, Kuniya Tanaka, Itaru Endo, Fritz C. Eilber, Robert M. Hoffman

**Affiliations:** ^1^ AntiCancer, Inc., San Diego, California, USA; ^2^ Department of Surgery, University of California, San Diego, California, USA; ^3^ Department of Gastroenterological Surgery, Graduate School of Medicine, Yokohama City University, Yokohama, Japan; ^4^ Division of Hematology-Oncology, University of California, Los Angeles, California, USA; ^5^ Department of Pathology, University of California Los Angeles, California, USA; ^6^ Division of Surgical Oncology, University of California, Los Angeles, California, USA; ^7^ Department of Pediatrics and Department of Orthopaedics, David Geffen School of Medicine, Mattel Children's Hospital, UCLA's Jonsson Comprehensive Cancer Center, University of California, Los Angeles, California, USA

**Keywords:** osteosarcoma, nude mouse, patient-derived xenograft, Salmonella typhimurium A1-R, tumor-targeting

## Abstract

Osteosarcoma occurs mostly in children and young adults, who are treated with multiple agents in combination with limb-salvage surgery. However, the overall 5-year survival rate for patients with recurrent or metastatic osteosarcoma is 20-30% which has not improved significantly over 30 years. Refractory patients would benefit from precise individualized therapy. We report here that a patient-derived osteosarcoma growing in a subcutaneous nude-mouse model was regressed by tumor-targeting *Salmonella typhimurium* A1-R (*S. typhimurium* A1-R, p<0.001 compared to untreated control). The osteosarcoma was only partially sensitive to the molecular-targeting drug sorafenib, which did not arrest its growth. *S. typhimurium* A1-R was significantly more effective than sorafenib (P <0.001). *S. typhimurium* grew in the treated tumors and caused extensive necrosis of the tumor tissue. These data show that *S. typhimurium* A1-R is powerful therapy for an osteosarcoma patient-derived xenograft model.

## INTRODUCTION

The tumor-targeting amino-acid-auxotrophic strain *Salmonella typhimurium* (*S. typhimurium*) A1-R is attenuated by auxotrophic mutations for Arg and Leu [1]. *S. typhimurium* A1-R has also been selected for high tumor virulence *in vivo*. *S. typhimurium* A1-R has been shown to be effective against all major types of human cancer in nude mouse models including: cancers of the prostate [1–3], breast [4–6], pancreas [7–11], and ovary [12, 13], as well as soft tissue sarcoma [14, 15] and glioma [16, 17]. *S. typhimurium* A1-R was also effective against high-grade osteosarcoma, including lung metastasis [18], breast-cancer brain metastasis [19], and experimental breast-cancer bone metastasis [20] in orthotopic mouse models of human cancer cell lines. *S. typhimurium* A1-R was also shown to be effective on pancreatic cancer stem cells [9], and in combination with anti-angiogenic agents [11]. *S. typhimurium* A1-R was also effective against cervical cancer [21], melanoma [22], soft-tissue sarcoma [14], and pancreatic cancer [10, 11] patient-derived orthotopic xenograft (PDOX) mouse models.

In recent studies, *S. typhimurium* A1-R was shown to be effective in a patient-derived orthotopic xenograft (PDOX) model of high-grade undifferentiated pleomorphic soft-tissue sarcoma (UP-STS) grown orthotopically in the right biceps femoris muscle of nude mice. Histological examination demonstrated eradication of the tumor treated with *S. typhimurium* A1-R followed by doxorubicin (DOX) [15].

*S. typhimurium* A1-R was also shown recently to be effective against a PDOX mouse model of follicular dendritic-cell sarcoma (FDCS) that was established in the biceps muscle of nude mice. The FDCS PDOX was resistant to both DOX and NVP-BEZ235 dactolisib (BEZ), but was sensitive to *S. typhimurium* A1-R [23].

Osteosarcoma occurs mostly in children and young adults [23, 24], who are treated with multiple agents in combination with limb-salvage surgery. However, the overall 5-year survival rate for patients with recurrent or metastatic osteosarcoma is 20-30%, which has not improved significantly over 30 years [25]. Refractory patients would benefit from precise individualized therapy.

In the present study, we used a patient-derived mouse xenograft model of osteosarcoma to demonstrate much higher efficacy of *S. typhimurium* A1-R administered by intratumor (i.t.) injection than the molecular-targeting drug, sorafenib.

## RESULTS AND DISCUSSION

### Comparison of the histology of the original patient tumor and mouse-grown patient tumor

**Figure 1:**
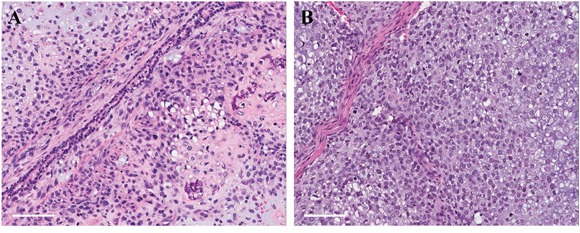
Hematoxylin and eosin (H&E) staining of original patient tumor and mouse grown tumor **A**. Original patient tumor (lung metastasis); **B**. Untreated patient tumor grown in nude mouse. Scale bars: 100 μm.

Both the original patient tumor (Figure 1A) and the mouse-grown patient tumor (Figure 1B) contained neoplastic chondroid matrix occupied by anaplastic cells. Both the original patient tumor and the mouse-grown patient tumor had hypercellular areas populated by anaplastic cancer cells displaying nuclear pleomorphism, coarse and hyperchromatic chromatin and abundant mitotic figures.

### Intra-tumor administration of *S. typhimurium* A1-R was highly-effective, in contrast to sorafenib, in a patient-derived osteosarcoma xenograft model

Nude mice were randomized into 3 groups: untreated control; treated group with sorafenib (10 mg/kg, p.o., 5 days a week, for 3 weeks); and treated with *S. typhimurium* A1-R (2.5×10^7^ colony forming units [CFU], by intra-tumor [i.t.] injection, weekly, for 3 weeks). All tumors were measured twice a week.

**Figure 2:**
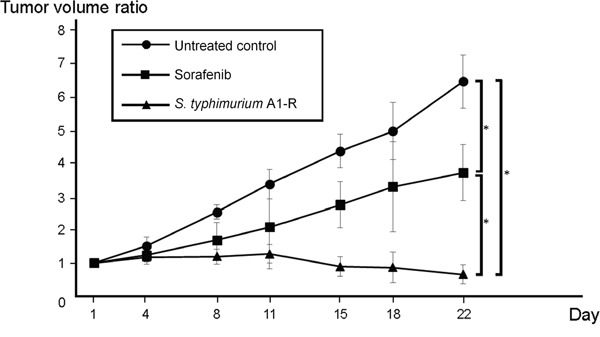
Intratumor (i.t.) administration of Salmonella typhimurium (S. typhimurium) A1-R regresses a patient-derived osteosarcoma xenograft model Eighteen subcutaneous tumors in nude mice were randomized into 3 groups: untreated control (n = 6), treated group with sorafenib (n = 6; 10 mg/kg, p.o., 5 days a week, for 3 weeks), and treated with S. typhimurium A1-R (n = 6; 2.5 × 107 colony forming units [CFU], intratumoral injection, weekly, 3 weeks). All tumors were measured twice a week and tumor volume was calculated using the following equation: Tumor volume (mm3) = tumor length (mm) × tumor width (mm) × tumor width (mm) ×1/2. Both sorafenib and S. typhimurium A1-R effectively reduced tumor growth. In addition, tumors treated with S. typhimurium A1-R achieved regression and were significantly smaller than sorafenib-treated tumors. Body weight was not lost in any mice. *P < 0.001. Error bars: ± 1 SD.

Sorafenib significantly reduced tumor growth (*P*<0.001), but the tumors continued to grow (Figure 2). In contrast, tumors treated with *S. typhimurium* A1-R regressed and were significantly smaller than sorafenib-treated tumors at the end of the study (*P*<0.001). Body weight range in all mice was from 26.6 to 31.1 g on day 1. There was no significant difference in body weight between the control group and treated groups at any time points.

### *S. typhimurium* A1-R growth in the treated tumors

**Figure 3:**
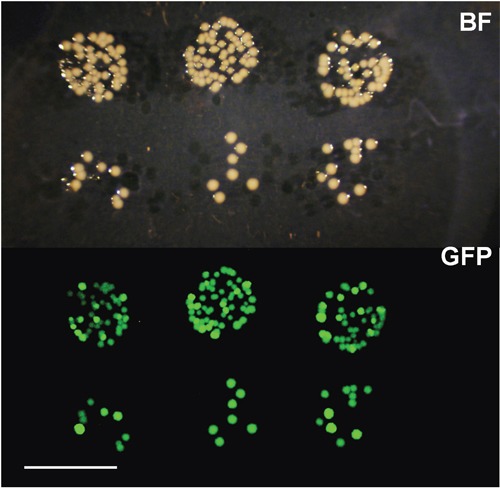
Culture of S. typhimurium from treated tumors Tumors were homogenized 48 hours after intra-tumor (i.t.) administration of S. typhimurium expressing GFP. Supernatants of the tumor homogenates were serially diluted and grown on agar medium for 12 hours and imaged with the OV100. Please see Materials and Methods. BF; bright field, GFP; green fluorescent protein. Scale bar: 10 mm.

*S. typhimurium*, expressing green fluorescent protein (GFP), was cultured in serial dilution from supernatants of tumor homogenates. Fluorescent bacteria were detected at all dilutions, indicating they were growing in the treated tumors (Figure 3).

### Effect of S. typhimurium on tumor histology

**Figure 4:**
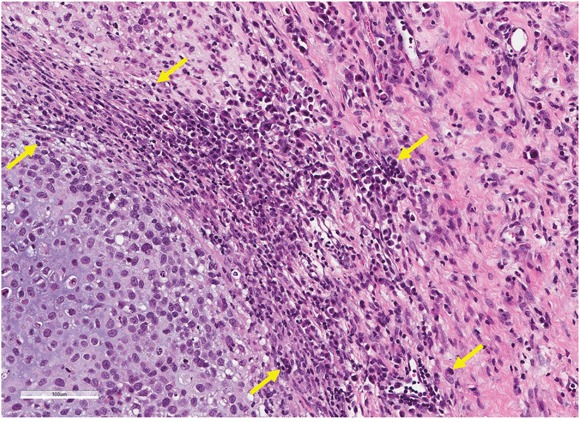
Effect of S. typhimurium on tumor histology Tumors were resected from nude mice at autospy, fixed in formalin, embedded in paraffin, sectioned and stained with hematoxylin and eosin (H&E) by standard methods. The figure shows the histology of an osteosarcoma treated with S. typhimurium A1-R. Necrotic areas are indicated by yellow arrows. Scale bar: 100 μm.

*S. typhimurium* caused extensive necrosis in the treated tumors as visualized in hematoxylin and eosin (H&E)-stained tumors (Figure 4).

Sorafenib, a multi-kinase inhibitor that blocks VEGFR, PDGFR, MAPK, and KIT, was previously shown to have efficacy against recurrent or un-resectable osteosarcoma as well as metastatic or recurrent angiosarcoma in Phase II clinical studies [26, 27].

*S. typhimurium* A1-R, in addition to being effective against the major types of human cancer in orthotopic nude mouse models [1–17, 21, 22], has been shown to be effective in patient-derived models of pancreatic cancer [10, 11], soft-tissue sarcoma [14, 15, 23] and melanoma [22]. In the present study, we now show that *S. typhimurium* A1-R could regress an osteosarcoma in a patient-derived model in contrast to sorafenib which could not.

Future experiments will examine efficacy of *S. typhimurium* A1-R against PDOX models of osteosarcoma and then in patients. Recently, the tumor-targeting obligate anaerobe *Clostridium novyi* NT has shown efficacy in leiomyosarcoma patients treated i.t. [28]. Bacterial therapy of cancer, after 80 years, has returned to the clinic [29].

Our group has developed many mouse models and treatment strategies for osteosarcoma [18, 30–38]. Bacterial therapy maybe the most efficacious.

Bacteria have important advantages for the treatment of cancer. Many bacteria naturally target tumors and they can be genetically-manipulated to improve selective tumor targeting and to reduce infection of normal tissue. Bacteria can directly kill infected cancer cells and possibly enhance immune effects against the tumor, even when tumors are drug resistant. Bacteria can grow in, and are not readily cleared from, infected tumors. In addition, bacterial targeting may not be limited by poor tumor vasculature [29].

Previously-developed concepts and strategies of highly-selective tumor targeting can take advantage of molecular targeting of tumors, including tissue-selective therapy which focuses on unique differences between normal and tumor tissues [39–44].

## MATERIALS AND METHODS

### Mice

Athymic *nu/nu* male nude mice (AntiCancer Inc., San Diego, CA), 4-6 weeks old, were used in this study. All animal studies were conducted with an AntiCancer Institutional Animal Care and Use Committee (IACUC)-protocol specifically approved for this study, which described the duration of the experiment, the frequency of animal monitoring, the survival aspects of the study, and any anticipated maximum tumor volume or weight loss thresholds at which animals would be euthanized, and in accordance with the principals and procedures outlined in the Guide for the Care and Use of Laboratory Animals, 8^th^ edition, under PHS Assurance Number A3873-1. Animals were anesthetized by subcutaneous injection of a 0.02 ml solution of 80-100 mg/kg ketamine, 10 mg/kg xylazine, and 3 mg/kg acepromazine maleate. Ibuprofen (7.5 mg/kg orally in drinking water every 24 hours for 7 days post-surgery) was used in order to provide analgesia post-operatively in the surgically-treated animals.

### Patient-derived tumor

The study was reviewed and approved by the UCLA Institutional Review Board (IRB #10-001857) before the study began. Written informed consent was obtained from the patient as part of the above-mentioned UCLA Institutional Review Board-approved protocol. A 16-year old patient with localized left distal femoral high-grade osteosarcoma underwent limb salvage distal femoral replacement. One year later, three bilateral metachronous pulmonary metastases appeared. The patient was treated with curative surgery at the Division of Surgical Oncology, University of California, Los Angeles (UCLA). The patient received chemotherapy using methotrexate, cisplatinum and doxorubicin (MAP) peri-operatively.

### Establishment of a mouse model of osteosarcoma by subcutaneous transplantation

A fresh sample of the osteosarcoma metastasized to the lung was obtained and transported immediately to the laboratory at AntiCancer, Inc., on wet ice. The sample was cut into 5 mm fragments and implanted subcutaneously in nude mice [15]. Implanted tumors were established in 4 weeks. The established tumor was cut into 5 mm fragments, then these fragments were implanted subcutaneously to the flank in nude mice for the treatment study.

### Preparation and administration of *S. typhimurium* A1-R

GFP-expressing *S. typhimurium* A1-R bacteria (AntiCancer, Inc., San Diego, CA, USA) were grown overnight on LB medium and then diluted 1:10 in LB medium. Bacteria were harvested at late-log phase, washed with PBS, and then diluted in PBS [1, 3, 4].

### Bacterial culture

To demonstrate bacterial viability in the treated tumor, a subcutaneous xenograft mouse model was used. Forty-eight hours after *S. typhimurium* A1-R i.t. injection (2.5 × 10^7^ CFU in 50 μl PBS), the treated tumor was homogenized, then suspended in PBS (phosphate-buffered saline, Corning, New York, NY). The suspension was serially diluted, then cultured in LB agar for 12 hours. GFP-expressing colonies of *S. typhimurium* A1-R were detected by the OV100 Small Animal Imaging System (Olympus, Tokyo, Japan) [45].

### Treatment protocol

Ten days after implantation, tumors reached 9 mm in diameter. Tumor-bearing mice were randomized into the following 3 groups of 6 mice each: G1, control without treatment (n=6); G2, treated with *S. typhimurium* A1-R (2.5 × 107 CFU in 50 μl PBS, i.t. injection), once a week, for 3 weeks (n=6); G3, treated with sorafenib (Selleckchem, Houston, TX, S7397), 10 mg/kg, p.o., 5 days a week, for 3 weeks (n=6). Tumor length, width and mouse body weight were measured twice in a week. Tumor volume was calculated with the following formula: Tumor volume (mm3) = length (mm) × width (mm) × width (mm) × 1/2. Data are presented as mean ± SD. When a tumor was not detectible, the tumor response was considered as complete remission. All treated mice were sacrificed on day 25, and tumors were resected for further histological evaluation [15].

### Histological examination

Fresh tumor samples were fixed in 10% formalin and embedded in paraffin before sectioning and staining. Tissue sections (5 μm) were deparaffinized in xylene and rehydrated in an ethanol series. Hematoxylin and eosin (H &E) staining was performed according to standard protocols. Histological examination was performed with a BHS System Microscope (Olympus Corporation, Tokyo, Japan). Images were acquired with INFINITY ANALYZE software (Lumenera Corporation, Ottawa, Canada) [15].

### Statistical analysis

SPSS statistics version 21.0 was used for all statistical analyses (IBM, New York City, NY, USA). Significant differences for continuous variables were determined using the Student's t-test. A probability value of P < 0.05 was considered statistically significant [15].

### Dedication

This paper is dedicated to the memory of A.R. Moossa, M.D. and Sun Lee, M.D.
